# Validity and bias on the online active Australia survey: activity level and participant factors associated with self-report bias

**DOI:** 10.1186/s12874-020-0896-4

**Published:** 2020-01-10

**Authors:** Rachel G. Curtis, Timothy Olds, Ronald Plotnikoff, Corneel Vandelanotte, Sarah Edney, Jillian Ryan, Carol Maher

**Affiliations:** 1 Alliance for Research in Exercise, Nutrition, and Activity, University of South Australia, GPO Box 2471, Adelaide, SA 5001 Australia; 2 Priority Research Centre for Physical Activity and Nutrition, The University of Newcastle, Callaghan, NSW 2308 Australia; 3 Physical Activity Research Group, School of Health, Medical and Applied Sciences, Central Queensland University, Rockhampton, QLD 4702 Australia

**Keywords:** Physical activity, Questionnaire, Accelerometry, Psychometrics

## Abstract

**Background:**

This study examined the criterion validity of the online Active Australia Survey, using accelerometry as the criterion, and whether self-report bias was related to level of activity, age, sex, education, body mass index and health-related quality of life.

**Methods:**

The online Active Australia Survey was validated against the GENEActiv accelerometer as a direct measure of activity. Participants (*n* = 344) wore an accelerometer for 7 days, completed the Active Australia Survey, and reported their health and demographic characteristics. A Spearman’s rank coefficient examined the association between minutes of moderate-to-vigorous physical activity recorded on the Active Australia Survey and GENEActiv accelerometer. A Bland-Altman plot illustrated self-report bias (the difference between methods). Linear mixed effects modelling was used to examine whether participant factors predicted self-report bias.

**Results:**

The association between moderate-to-vigorous physical activity reported on the online Active Australia Survey and accelerometer was significant (*r*_*s*_ = .27, *p* < .001). Participants reported 4 fewer minutes per day on the Active Australia Survey than was recorded by accelerometry (95% limits of agreement −104 – 96 min) but the difference was not significant (*t*(343) = −1.40, *p* = .16). Self-report bias was negatively associated with minutes of accelerometer-recorded moderate-to-vigorous physical activity and positively associated with mental health-related quality of life.

**Conclusions:**

The online Active Australia Survey showed limited criterion validity against accelerometry. Self-report bias was related to activity level and mental health-related quality of life. Caution is recommended when interpreting studies using the online Active Australia Survey.

## Background

Accurate assessment of physical activity is imperative for surveillance of population health behaviours and to examine the efficacy of physical activity interventions. Self-report questionnaires are a low-burden and cost-effective method of assessment and are used widely in epidemiological and experimental research [1]. In particular, the internet has become a convenient platform for survey-based data collection. Despite the advantages of using self-report measures of activity, they are prone to bias due to factors including inaccurate memory and social desirability [2]. Potential bias can be examined by comparing self-report activity with activity estimated using device-based measures such as accelerometry. On average, self-report measures have produced higher estimates of activity than direct measures such as accelerometry and heart rate monitoring [2, 3]. Using different measures of activity could lead to different conclusions about population disease risk or the efficacy of physical activity interventions. It is therefore important to choose a reliable and valid measure that is appropriate for the sample. The Active Australia Survey (AAS) is a popular 8-item measure of activity that asks participants to report the frequency and duration of physical activity in the past week, including walking, gardening, moderate and vigorous activity [4, 5]. Initially designed to be administered via telephone or face-to-face interview [5], the AAS is administered as a self-report paper-based or online questionnaire. Preliminary research, outlined below, suggests the online AAS may be unreliable. Further research is required to confirm this finding. Additionally, key questions remain about the potential sources of variance in the association between the AAS and physical activity measured directly.

Research has demonstrated that the telephone-administered AAS has acceptable criterion validity compared to device-measured moderate-to-vigorous physical activity (MVPA). For example, among university staff and students, MVPA measured via the AAS was strongly associated with MVPA measured via accelerometer (*r*_*s*_ = .61) [6]. In addition, paper-based versions of the AAS have shown moderate reliability against accelerometry in middle-aged American (*r*_*s*_ = .40) [7] and Australian women (*r*_*s*_ = .52) [8] and sedentary middle-aged adults (*r*_*s*_ = .49–.65) [9]. However, among Australian government employees, the association between AAS- and accelerometer-derived MVPA was lower when the AAS was administered online (*r*_*s*_ = .47–.57) than when administered via telephone (*r*_*s*_ = .65) [10]. Furthermore, a recent study found little evidence to support the validity of the online AAS compared to accelerometry (MVPA *r*_*s*_ = .23) among adults enrolled in a physical activity randomised controlled trial (RCT) [11]. Additional research should examine whether the AAS is a valid measure of physical activity when administered online.

Although considerable literature has evaluated the association between AAS- and accelerometer-recorded MVPA, little research has examined participant factors that might affect reporting on the AAS. Among university staff and students, the difference in MVPA minutes between the telephone-administered AAS and accelerometry was positively associated with minutes of MVPA measured as the average of the two measures (β = 0.50, SE 0.11) [6]. Recall may be more difficult when there is more activity to remember, particularly if the activity is not routine. In contrast, associations between online AAS- and accelerometer-derived activity did not differ based on RCT participants’ accelerometer-measured MVPA level (= < 150 min *r*_*s*_ = .17; > 150 min *r*_*s*_ = .25) [11]. Thus, the relationship between participants’ physical activity level and self-report bias on the online AAS remains unclear.

Other participant factors could also be associated with self-report bias on the AAS. Among RCT participants, associations between online AAS- and accelerometer-derived vigorous activity were lower for men and older adults [11]. Additionally, associations between AAS- and accelerometer-derived moderate activity were lower for overweight compared to healthy weight participants [11]. Although this study showed that associations between AAS- and accelerometer-derived activity did not differ based on education [11], previous research has shown stronger associations between MVPA reported on the International Physical Activity Questionnaire and accelerometry among more highly educated participants [12]. Additionally, adults with a more positive general health perception have shown greater overestimation of activity in the past year (i.e., extremely active/moderately active/not very active) compared to activity classified using 4 days of heart rate monitoring [13]. A more comprehensive understanding of these potential correlates of self-report bias is important for researchers interpreting results of previous research using the online AAS and considering whether the online AAS might be an appropriate measure of physical activity in their sample.

In sum, limited research has examined the validity of the online administration of the AAS. This study therefore adds to the literature by examining the association between MVPA reported on the online AAS and recorded via accelerometer in a new sample. Additionally, it extends previous research by examining self-report bias (the difference between daily minutes of MVPA reported on the online AAS and daily minutes of MVPA derived from accelerometry). While previous research has examined the association between measurement methods in different subgroups [11], this is the first study to examine predictors of bias on the online AAS. The objectives of this study were to (1) evaluate the criterion validity of minutes of MVPA on the online AAS relative to accelerometry by examining both the association between methods and the difference between daily minutes of MVPA reported on the online AAS and daily minutes of MVPA derived from accelerometry, and (2) examine whether participant factors (activity level, age, sex, education, body mass index (BMI) and general health perception) were associated with bias. Both physical and mental health-related quality of life were included, as general health perception reflects both physical and mental health [14].

## Methods

### Participants and design

This study used data from an RCT evaluating the effectiveness of an mHealth physical activity intervention, “Active Team”. Active Team is a purpose-built smartphone app that uses gamification and social features to encourage inactive adults to engage in a minimum of 150 min of MVPA per week. The recruitment and intervention protocol has been described elsewhere in detail [15]. Briefly, participants were recruited through Facebook and media recruitment campaigns and were eligible to participate if they were aged 18 to 65 years, used Facebook at least weekly, were fluent in English, lived in Australia, reported completing less than 150 min of MVPA per week, and were able to form a team with two to seven of their existing friends and family members. Teams were randomly allocated to either the waitlist control condition, basic experimental condition (pedometer plus a basic version of the app with no social and gamification features), or socially-enhanced experimental condition (pedometer plus the app with social and gamification features). Participants completed a survey and accelerometry assessment at baseline, 3 months and 9 months. This study used cross-sectional data from 344 participants who completed the survey and accelerometry assessment at 3 months because the survey and accelerometry assessments were completed closer together at 3 months than at baseline (participants were requested to complete both assessments within 3 weeks, which was not a requirement at baseline). Assessment times varied; 31% completed the AAS (which assesses the previous 7 days) after day 7 of the accelerometry assessment (median 11 days, IQR 5–20) and 69% completed the AAS before day 7 of the accelerometry assessment (median 13 days, IQR 8–17). The trial is registered with the Australian and New Zealand Clinical Trial Registry (ACTRN12617000113358). Ethical approval was obtained from the Human Research Ethics Committee of the University of South Australia. Participants provided informed consent prior to commencing the study.

### Measures

#### Accelerometry

Participants were asked to wear a GENEActiv accelerometer (Activinsights Ltd., UK) for 24 h per day for 7 days, except during water-based activities such as swimming and showering. Activity was measured continuously at 50 Hz. Moderate and vigorous activity were classified for each 60-s epoch based on established thresholds [16]. MVPA was calculated as the average daily minutes of moderate and vigorous activity recorded during activity bouts (defined as activity of 10 min or more, allowing for 20% of activity counts to be below the threshold for moderate activity, in keeping with previous research [6, 8, 12]). Data were considered valid if the accelerometer was worn for at least 10 h whilst awake on at least 4 days, including a minimum of 1 weekend day [17]. Periods of 60 min of consecutive counts less than 25 were considered non-wear time. Participants had valid accelerometry data for a median of 7 days (IQR 6–7) and wore the accelerometer for a median of 16.9 h per day (IQR 16.0–17.7). Once sleep logs were included for participants who did not wear the accelerometer overnight, data summed to 23.7 h (IQR 23.5–24.0), indicating there was little missing activity data. The GENEActiv has shown excellent reliability (CVintra =1.4%, CVinter =2.1%) and validity (*r* = .98) using a mechanical shaker, as well as excellent criterion validity when worn on the left wrist, using relative VO_2_ as the criterion (*r* = .86) [16].

#### Active Australia survey

Participants completed the AAS by reporting the number of times and total minutes they spent in the previous week (1) walking continuously for at least 10 min, (2) doing vigorous gardening or heavy work around the yard, (3) doing vigorous physical activity which made them breathe harder or puff and pant, and (4) doing other more moderate physical activities [5]. Weekly MVPA was calculated as the sum of minutes spent walking, in moderate activity, and in vigorous activity. This follows the standard method for calculating total activity [5], except that vigorous activity was not weighted to enable comparison with accelerometer-recorded MVPA minutes. Daily MVPA was calculated as weekly MVPA divided by 7.

#### Potential predictors

Further potential predictors of self-report bias on the online AAS were chosen based previous research suggesting possible differences in the association between self-report and device-derived activity according to age, sex, BMI [11], education [12], and general health perception [13]. Predictors included age, sex, education (high school or less, technical or further education institution, or university degree), BMI (kg/m^2^ calculated from self-reported weight and height) and physical and mental health-related quality of life [12-Item Short Form Health Survey (SF-12) standardised to US population norms [18, 19]]. The SF-12 has been shown to have high 2-week test-retest reliability (physical health *r* = .89; mental health *r* = .76) and to be highly correlated with the SF-36 (physical health *r* = .95; mental health *r* = .97) [19].

### Analysis

To examine objective 1 (criterion validity of the online AAS), a Spearman’s rank coefficient described the association between AAS-derived and accelerometer-derived MVPA minutes. Spearman’s coefficient was used because physical activity data were not normally distributed, and a bivariate scatterplot indicated a monotonic but potentially non-linear association. Spearman’s rho has been used widely in validation studies, including previous studies of the AAS [6–11]. Bias scores were calculated for each participant as AAS-reported - accelerometer-recorded MVPA. A Bland-Altman plot presented all bias scores and indicated the mean bias and the limits of agreement (±1.96*SD*; an interval within which 95% of the bias scores lie) [20]. Bias scores, which were approximately normally distributed, were plotted against the average of the two measures as a proxy for the ‘true’ level of MVPA (as per the traditional Bland-Altman approach, because accelerometry is not without measurement error). A one sample t-test examined whether the mean bias score was significantly different from 0.

To examine objective 2 (predictors of bias on the online AAS), linear mixed effects modelling predicted bias scores. Continuous scores for the predictors were centred on the sample mean. Model 1 examined whether bias was predicted by minutes of accelerometer-recorded MVPA. Model 2 additionally included age, sex and education. Model 3 examined all predictors, additionally including BMI, and physical and mental health-related quality of life. To account for structure of the data (persons nested within teams), team was included as a random effect. The full information maximum likelihood estimator was used to enable inclusion of incomplete cases by estimating parameters using all available data points (note that missing data was minimal: 3 participants had missing data for BMI, with 2 of these participants also missing data for age) [21]. Model fit was indicated by the log likelihood (−2LL), Akaike Information Criterion, and Bayesian Information Criterion, with lower values demonstrating better fit [22]. Fit indices were inspected to confirm the inclusion of additional parameters (i.e. in models 2 and 3) did not considerably reduce model fit. Analyses were completed using SPSS 25.

## Results

Participants tended to be female, young or middle-aged, overweight and highly educated, and recorded an average of 41 min of MVPA per day via accelerometer (Table 1). BMI and physical health-related quality of life were associated with accelerometer-derived MVPA, whereas sex and mental health-related quality of life were associated with self-report MVPA.

**Table 1:** Participant descriptive statistics and correlations^*a*^

Variables		2	3	4	5	6	7	8
1. Age (years), *M* (*SD*)	41.9 (11.6)	.02	.10	.22**	−.10	.22**	.11	−.13*
2. Female, *n*(%)	258 (75.0)	–	.12*	−.01	.08	.02	.11*	.08
3. Highest education level			–	.10	.01	−.06	.08	.08
High school or less, *n*(%)	51 (14.9)							
Technical or further education, *n*(%)	103 (30.1)							
University degree, *n*(%)	188 (55.0)							
4. BMI, *M* (*SD*)	29.7 (6.8)			–	−.28**	−.01	−.11	−.33**
5. Physical health QoL, Mdn (IQR)	48.4 (41.4–52.7)				–	−.07	.06	.27**
6. Mental health QoL, Mdn (IQR)	49.3 (43.4–53.8)					–	.29**	.07
7. AAS MVPA min/day, Mdn (IQR)	34 (18–68)						–	.27**
8. Accelerometer MVPA min/day, Mdn (IQR)	41 (21–73)							–

*BMI* body mass index, *QoL* Quality of life, *MVPA* moderate-to-vigorous physical activity, *AAS* Active Australia Survey

^a^Spearman’s rho, except for point biserial for correlations involving sex, and Pearson’s for the correlation between age and BMI

*p < .05, **p < .001

**Fig. 1:**
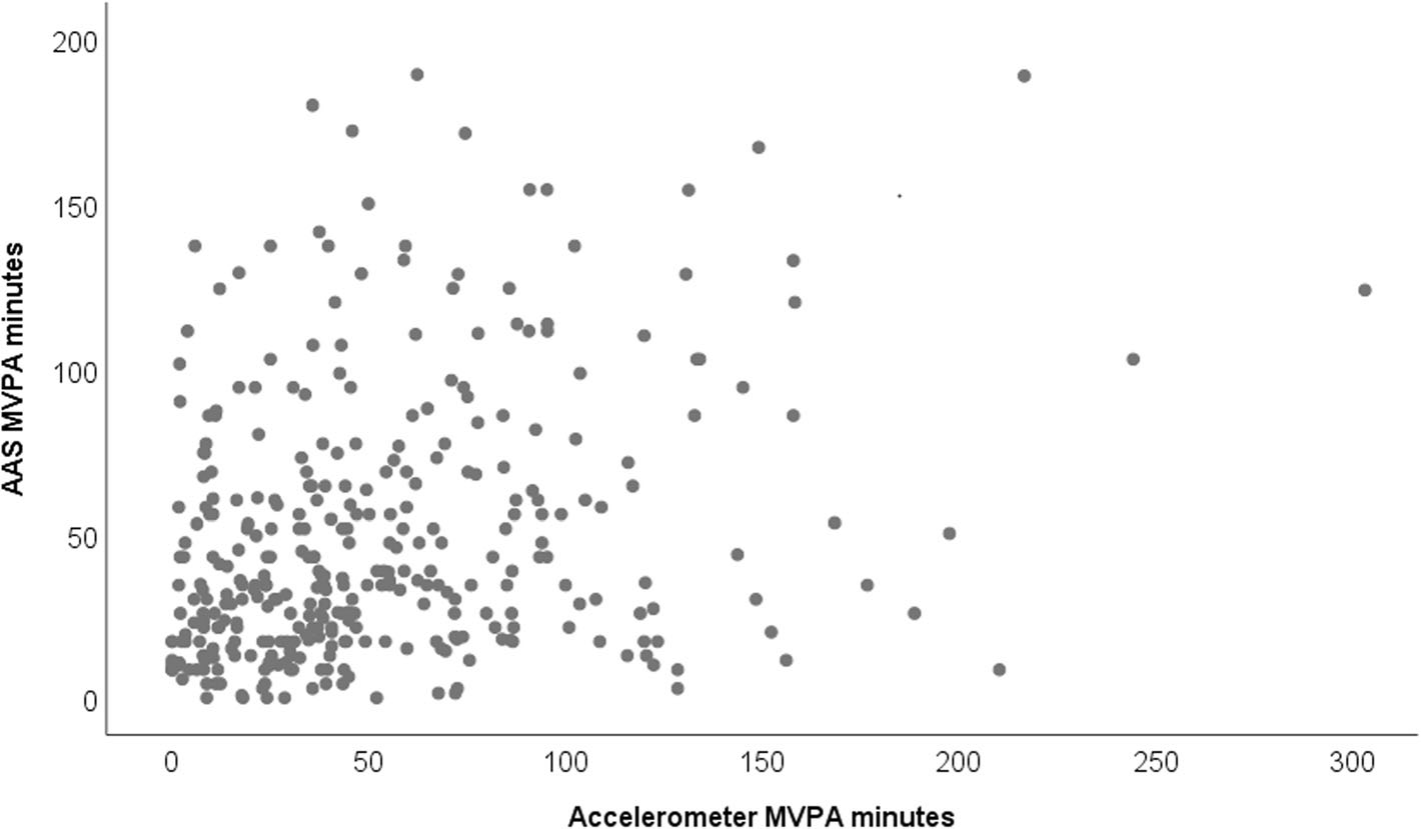
AAS-reported and accelerometer-recorded daily minutes of MVPA. AAS = Active Australia Survey, MVPA = moderate-to-vigorous physical activity

**Fig. 2:**
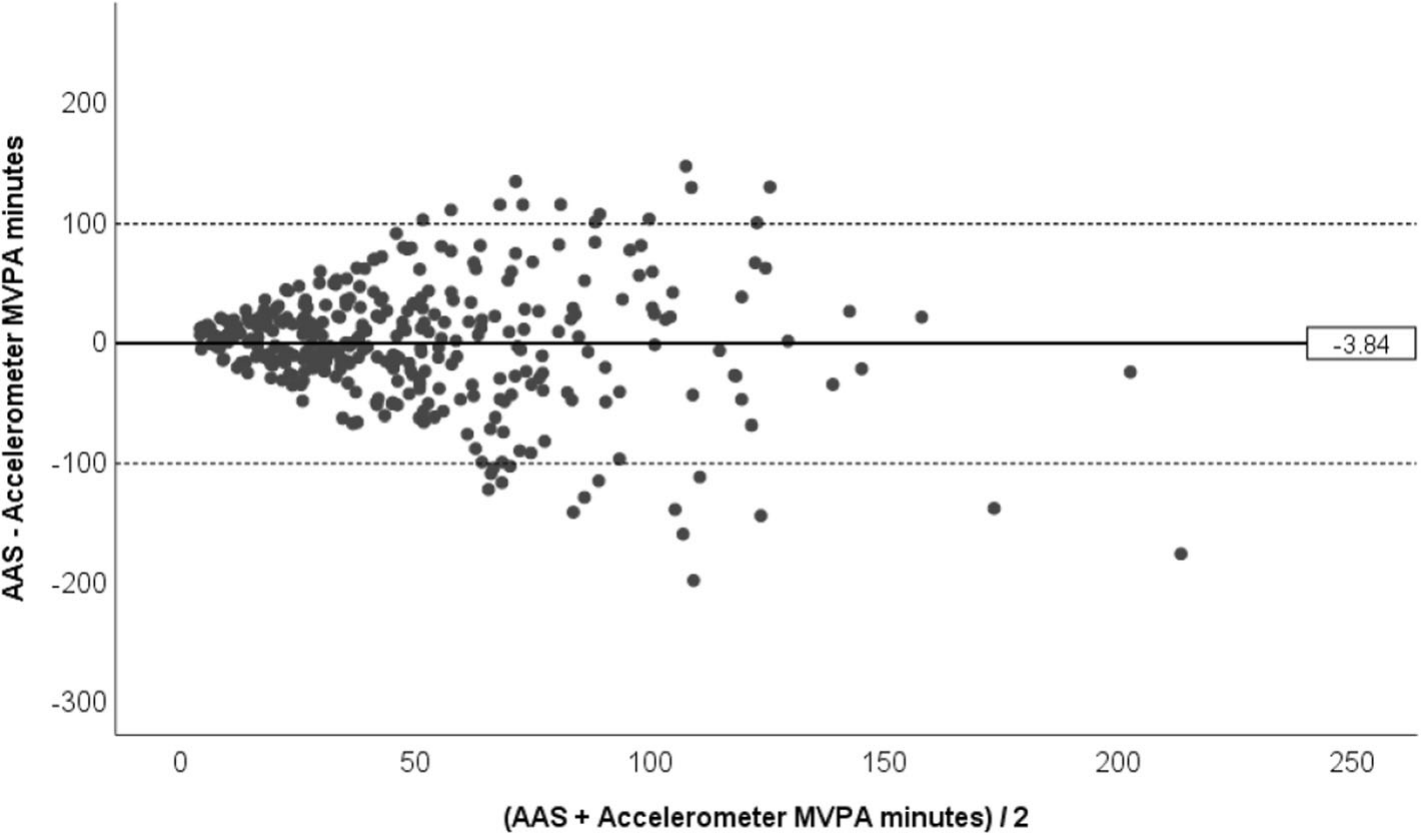
Mean difference between AAS-reported and accelerometer-recorded daily minutes of MVPA. AAS = Active Australia Survey, MVPA = moderate-to-vigorous physical activity

With regards to objective 1, the association between AAS- and accelerometer-derived MVPA (see Fig. 1) was .27 (*p* < .001). Figure 2 illustrates the mean difference and limits of agreement (±1.96*SD*) between daily minutes of moderate-to-vigorous activity reported on the Active Australia Survey and moderate-to-vigorous activity measured via accelerometry. On average, participants reported 4 fewer minutes of MVPA per day on the AAS (*M* = −3.8, *SD* = 51.1) than was recorded by accelerometry, with the limits of agreement ranging from −104 to 96 min. The mean bias score was not significantly different from 0 (*t*(343) = −1.40, *p* = .16). The plot also indicates a greater range in bias scores at higher minutes of MVPA and a possible trend whereby participants with high levels of MVPA reported relatively lower MVPA on the AAS.

With regards to objective 2, Table 2 shows results from the linear mixed models examining predictors of bias on the AAS. Higher accelerometer-recorded MVPA was associated with more negative bias scores in all models. Older age was associated with more positive bias scores in Model 2, but not when BMI and health-related quality of life were included (Model 3). Greater mental health-related quality of life was associated with more positive bias scores (Model 3). Sex, education, BMI and physical health-related quality of life were not associated with self-report bias.

**Table 2:** Predictors of the difference between AAS-reported and accelerometer-recorded daily minutes of MVPA

Variables	Model 1		Model 2		Model 3	
	*B* (SE)	β	*B* (SE)	β	*B* (SE)	β
Intercept	−3.88 (2.22)		3.54 (6.24)		5.86 (6.07)	
Accelerometer MVPA min/day	−0.75 (0.05)**	−0.66**	−0.75 (0.05)**	−0.66**	−0.79 (0.05)**	−0.69**
Age			0.38 (0.18)*	0.09*	0.24 (0.18)	0.05
Female			−6.92 (4.76)	−0.14	−6.54 (4.66)	−0.13
TAFE^*a*^			0.31 (6.54)	0.01	−1.54 (6.41)	−0.03
University degree^*a*^			−4.20 (6.10)	−0.08	−7.60 (5.98)	−0.15
BMI					−0.10 (0.33)	−0.01
Physical health QoL					0.26 (0.25)	0.05
Mental health QoL					0.89 (0.20)**	0.18**
Intercept variance	101.68 (83.04)		86.59 (82.45)		10.48 (74.84)	
Residual	1360.54 (125.57)		1346.04 (125.49)		1342.73 (126.40)	
-2LL	3481.40		3454.60		3426.39	
AIC	3489.40		3470.60		3448.39	
BIC	3504.76		3501.28		3490.54	

*MVPA* Moderate-to-vigorous physical activity, *AAS* Active Australia Survey, *TAFE* Technical or further education institutions, *BMI* body mass index, *QoL* Quality of life, *LL* log likelihood, *AIC* Akaike Information Criteria, *BIC* Bayesian Information Criteria

^a^High school as the reference category

*p < .05, **p < .001

To demonstrate the effects of accelerometer-derived MVPA and mental health-related quality of life on bias scores, relevant values were substituted into the linear equation of Model 4 to predict scores for females with a university degree at the mean of the remaining covariates. The model predicted that those who recorded 8 min of MVPA per day via accelerometer (*M*-1*SD*) overreported their daily MVPA on the AAS by 27 min. In contrast, those who recorded 52 (*M*) and 97 min (*M* + 1*SD*) of MVPA via accelerometer underreported their MVPA on the AAS by 8 and 44 min, respectively. Furthermore, those with low (*M*-1*SD* = 38) and average (*M* = 48) scores for mental health-related quality of life underreported their daily MVPA on the AAS by 17 and 8 min, respectively, whereas those with high scores for mental health-related quality of life (*M* + 1*SD* = 58) showed no bias.

## Discussion

The association between AAS- and accelerometer-derived MVPA was weak. Although there was no mean difference between assessment methods, the limits of agreement were wide. Examination of the criterion validity of the online administration of the AAS relative to accelerometry (objective 1) therefore suggests that caution should be exercised when interpreting results from studies using the online AAS. The AAS may be less reliable when administered online because participants may progress through the survey quickly and respond with less consideration than they would during an interview. In addition, the interview format allows participants to clarify the meaning of questions and enables interviewers to ensure values are plausible and activities are classified correctly [5]. It is less clear why the online version might be less reliable than the paper-based version.

Examination of bias (objective 2) showed significant results. Accelerometer-recorded minutes of MVPA was negatively associated with reporting bias. The positive bias (potential overreporting) demonstrated by participants with low activity levels may be due to social desirability (responding in a manner believed to be viewed more favourably), which has been shown to predict overreporting physical activity [23]. The negative bias (potential underreporting) demonstrated by participants with high accelerometer-recorded activity levels may be due to higher levels of occupation or transport activity that was not purposeful and therefore not recalled. Previous research has shown the International Physical Activity Questionnaire is less reliable for participants who spend proportionally greater time on occupational physical activity and cycling for transport [12]. Alternatively, very active participants may be relatively more fit and therefore interpret more moderate activities as being of lighter intensity and not reportable. If so, this finding would be in contrast to a study that found that more active participants showed a more positive reporting bias on the telephone AAS [6]. This also contrasts with the previous finding that the association between online AAS- and accelerometer-derived activity did not differ based on whether participants met physical activity guidelines [11]. Notably, these studies used less active participants and different accelerometers, which could account for a difference in activity estimates.

Mental health-related quality of life was positively associated with reporting bias. Predictions showed that participants with low scores on the SF-12 mental health scale tended to report lower activity on the AAS relative to accelerometery. This could perhaps reflect a general tendency for more negative responding. Interestingly, age was not associated with reporting bias once health-related quality of life was included Model 3 (Table 2). This is likely because age and mental health-related quality of life were moderately positively correlated (Table 1).

Online self-report measures of activity such as the online AAS are low-burden, cost-effective, and, unlike accelerometry, can provide contextual information about the types of activities participants have done. Despite showing lower validity, self-report measures may therefore be the most appropriate choice for some studies. To facilitate the use of higher-quality self-report measures, research should therefore examine methods of administration that may improve reporting on the online AAS, particularly for participants characterised by more extreme (high or low) physical activity levels or low mental health-related quality of life. Providing more detailed written examples of activity at different intensities could improve participants’ classification of their activities. One study found that reporting on the online AAS was improved when participants were presented with video cues that showed adults participating in moderate- and vigorous-intensity activities [10]. This appears to be a promising method, and relatively easy to apply in online environments, that warrants further attention. In addition, it may be useful to examine whether reliability improves after excluding data from participants with unusually short completion times.

### Strengths and limitations

Strengths of this study include a large sample and that the accelerometry assessment measured multiple days (median 7 days) and included both weekdays and weekend days; however, limitations must be noted. This study compared the online AAS to accelerometry as a reference measure, but accelerometry cannot measure activity without error. The use of different accelerometers with different body placements, thresholds or epochs lengths can result in different estimates of activity [17, 24]. In particular, although wrist-worn accelerometers may be preferred due to higher compliance [25, 26], wrist-worn accelerometers tend to be less reliable than hip-worn accelerometers [27]. This may explain why this study found a weaker association using wrist-worn GENEActiv accelerometers than a previous study using a hip-worn Actigraph accelerometers [10]; though similarly weak associations were found in an additional study using hip-worn Actigraph accelerometers [11]. Additionally, the accelerometer could not be worn during water-based activities. Research comparing the online AAS to different direct measures of activity may therefore be useful to further establish validity. Furthermore, participants did not complete the AAS directly after the accelerometer assessment. Nonetheless, the analysis can be considered valid as MVPA has been shown to be relatively stable in adults over periods of 1 to 4 weeks (ICC 0.89–0.90) [28]. In addition, the number of days between assessments was not correlated with bias scores (*r*_*s*_ = .02, *p* = .66). The sample consisted primarily of wealthy, overweight women who were born in Australia, therefore the findings of this study may not generalise as well to other populations. Research should examine the validity of the online AAS in more diverse samples.

## Conclusion

In sum, this study showed that the online AAS has limited criterion validity compared to accelerometry, although more research is needed. The association between the online AAS and accelerometry varied according to physical activity level and mental health-related quality of life. Methods to improve reporting on the online AAS may therefore be required. To obtain a more comprehensive understanding of the potential usefulness of the online AAS, future research should examine its reliability and validity against alternative measures of activity, using more diverse samples.

## Data Availability

The datasets used and/or analysed during the current study are available from the corresponding author on reasonable request.

## Abbreviations

AAS: Active Australia Survey
BMI: body mass index
MVPA: moderate-to-vigorous physical activity
